# The Effect of Claustrophobic Tendencies on Digital Spatial Preferences

**DOI:** 10.3389/fpsyg.2022.874765

**Published:** 2022-06-23

**Authors:** Sorim Chung

**Affiliations:** Department of MIS, Marketing, and Analytics, Rochester Institute of Technology, Rochester, NY, United States

**Keywords:** claustrophobia, need for space, spatial constraints, spatial perception, digital interface

## Abstract

In digital environments, the demand for larger devices (e.g., larger smartphones) has been growing continuously, indicating users’ spatial needs in digital interfaces. This study explores the need for space in digital interfaces in relation to claustrophobic tendencies. The findings from two studies consistently report that (1) stronger claustrophobic tendencies toward physical spatial constraints are positively associated with a stronger need for digital space. The results also demonstrate that (2) people with elevated claustrophobic tendencies and a stronger need for digital space perceive stronger spatial constraints on digital interfaces, and (3) claustrophobic tendencies and need for digital space have stronger effects on spatial constraints with a more complex grid design. Interestingly, the findings suggest that (4) claustrophobic tendencies are more closely associated with spatial needs from attentive tasks (e.g., reading a long document), than device-related spatial needs (e.g., large screen preferences), implying that such claustrophobic tendencies are more likely to influence cognitive tasks on digital devices. Overall, the findings indicate that claustrophobic tendencies may be utilized beyond medical purposes and may assist researchers and business practitioners understand users’ spatial needs in fast-changing digital environments.

## Introduction

Space is an important design element not only for physical environments but also for digital platforms. In the digital environment, the size of one’s space is often determined by the user’s device type, and the demand for larger devices has been growing continuously. According to a survey, 98% of respondents preferred using multiple monitors for computer tasks (Owens et al., 2012), and over 60% used larger devices, such as PCs, to visit a website (Szymkowiak and Garczarek-Bąk, 2018).

The demand for larger devices is similar in the smartphone market. Fifty percent of smartphone owners cite screen size as one of the top three reasons for purchasing a new smartphone, with 24% of them citing it as the primary reason (Nielsen, 2015). Since the launch of the first iPhone, consumers have expressed a strong desire for more screen space for convenience and ease of use (Pinola, 2022), and the demand is more apparent now as they perform more tasks on smartphones, ranging from simply texting and making phone calls to shopping and navigating maps. The strong demand has also led to an increase in smartphone sizes over the decade; larger screen size has become a key feature, heavily promoted by smartphone manufacturers, such as Apple and Samsung, over the last few years (Velazco, 2021).

Besides the popularity of larger devices, researchers have also addressed the important role that spatial cues play in people’s visual information processing (VanRullen, 2003) and overall user experiences (Eroglu et al., 2001; Kim and Kim, 2020). The literature suggests that individuals differ in how they process their spatial needs (Radomsky et al., 2001) and visual experiences (Krishna, 2010; Windey and Cleeremans, 2015), and those who are particularly more susceptible to visual–spatial constraints (e.g., confined physical surroundings) have been medically diagnosed with claustrophobia (Öst, 2007). Despite the important role of spatial needs in information processing, it is unknown whether similar spatial needs exist for digital interfaces (e.g., websites). Most studies on digital user experiences have focused on examining the spatial perceptions (e.g., perceived crowding or clutter) associated with design elements, such as text spacing or grids (Couper et al., 2013; Galliussi et al., 2020). Not to mention, no research has examined whether those with elevated claustrophobic tendencies (sensitive to *physical* confinement) will lead to similar spatial discomfort in *digitally* confined settings.

To remedy this shortcoming, this study explores whether claustrophobic tendencies in physical surroundings translate into a preference for space in digital interfaces. Additionally, it discusses whether the need for digital space influences perceived spatial constraints on a digital interface. The study presents novel findings that claustrophobic tendencies are associated with the need for larger digital space and perceived spatial constraints. The findings assist researchers and business practitioners by providing deeper insights into digital users’ visual and spatial experiences in the current (e.g., web-based interfaces) and future digital environments (e.g., Metaverse).

## Literature Review

### Claustrophobia

The term claustrophobia refers to “*an exaggerated fear of closed places, such as closets, subways, tunnels, telephone booths, elevators, small rooms, crowds, or other enclosed or confined spaces*” (Doctor et al., 2008, p. 137). Medically, claustrophobia has been used to diagnose excessive anxiety triggered by spatial constraints in physical spaces and is classified as one of the situational phobias in the recent edition of the Diagnostic and Statistical Manual of Mental Disorders (DSM-5-TR) and International Classification of Disease (ICD-10-CM; American Psychiatric Association, 2022). A few tools that are designed to specifically measure the level of claustrophobia include Radomsky et al.’s (2001) Claustrophobia Questionnaire (CLQ) and a slightly shorter version of the Claustrophobia Scale by Öst (2007).

Among the general population, only 12.5% are diagnosed with claustrophobia (Vadakkan and Siddiqui, 2021), but many individuals exhibit claustrophobic tendencies without being diagnosed (Kirkpatrick, 1984); people experience increased stress in crowded environments (Hui and Bateson, 1991; Eroglu et al., 2005; Ali et al., 2021). Retail crowding leads to lower customer satisfaction and shopping duration (Hui and Bateson, 1991; Eroglu et al., 2005) and reduces customers’ willingness to pay for in-store products (O’Guinn et al., 2015). Crowding and visual complexity are also associated with poor shopping experiences (Machleit et al., 2000; Eroglu et al., 2005), increased distractions (Hock and Bagchi, 2018), reduced ability to process information (Hock and Bagchi, 2018), and purchase intentions (Esmark and Noble, 2016).

### Technological and Design Claustrophobia

For spatial needs in the digital space, researchers have focused primarily on examining the spatial constraints in the context of crowding (Dobres et al., 2018) and visual complexity of digital interfaces (Sohn et al., 2017) rather than claustrophobic tendencies. Specifically, they have assessed perceived constraints based on perceived clutter (Lee and Cho, 2010; Lawrance and Mouliason, 2013; Ordenes et al., 2019), perceived crowding (Dobres et al., 2018), and differences in content design, such as spacing between text (Slattery et al., 2016; Galliussi et al., 2020) or grid complexity (Couper et al., 2013), regardless of individual differences in claustrophobia.

Despite the paucity of research, some researchers have conceptually proposed claustrophobic responses in the digital space. O’Reily (2011) used the term “*technological claustrophobia*” to describe “*outward-bound confinement*” (p. 7) triggered by digital spaces that are completely virtual without any sense of tangible surroundings, such as touch or smell. O’Reily argued that due to the way the digital space is systematized by an electronic framework, people tend to develop a “*desire to escape*” from this fabricated virtual world, even though the digital space was designed to “*externalize*” what was originally considered as the internal surroundings of the physical world (O’Reily, 2011, pp. 3–5). Similar definitions were later introduced in other publications. Alec Maasen, a multimedia artist, used the term “claustrophobia” to describe the anxiety caused by immersive experiences *via* digital devices (Massen, 2015). Christol (2016) also used a similar term “*design claustrophobia*” to describe the visual anxiety disorder onset by “*certain stimuli or situations, such as too much copy, a complex message, multiple messages being shared at the same time, tight spaces, not enough white space, no room to breathe or rest, bad fonts, overwhelming ink consumption, digital clutter,* etc.*”* (Christol, 2016).

### Digital Spatial Constraints

The level of digital spatial constraints is often determined by broad factors that include overall experiences (Kim et al., 2007) and ease of use (Yu and Kong, 2016), as well as specific design components, such as the grid structure (Couper et al., 2013), paragraph length, font, typeface spacing (Miniukovich et al., 2017), and the number of hyperlinks (Lawrance and Mouliason, 2013).

Similar to physical settings, digital spatial constraints also work adversely on users’ overall experiences. For instance, digital density has negative impacts on affective and behavioral outcomes (Baker and Wakefield, 2012), customer satisfaction (Sohn et al., 2017), and emotion (Mosteller et al., 2014). Negative attitudes triggered by online retail cues, including spatial constraints, decrease approach behaviors, such as store re-patronage (i.e., website revisit), amount of money and time spent in a store, and search for store offerings (Eroglu et al., 2001). On the contrary, sufficient spacing in digital interfaces improves user experiences (Liu et al., 2017), while excessive space negatively affects reading due to a larger visual span that requires more scrolling (Yu et al., 2007). In other words, spacious interfaces positively influence user experiences as long as it involves an adequate amount of space.

Therefore, to optimize digital interface design, it is important to understand individual differences in spatial needs, which may be associated with one’s claustrophobic tendencies. Although claustrophobic tendencies have been primarily measured to understand individuals’ sensitivity toward *physical* spatial constraints, I believe that they can be also used to predict the level of spatial needs in a *digital* context. People with an elevated claustrophobic tendency may display a preference for larger screen spaces and stronger perceived spatial constraints. Thus, I propose:

*H1: An elevated claustrophobic tendency in physical environments will lead to a greater need for space in digital environments.*

*H2: An elevated claustrophobic tendency and a stronger need for digital space will lead to stronger spatial constraints, and the effects would be further amplified while viewing spatially constrained content.*

## Study 1: Claustrophobic Tendency and Digital Spatial Needs

The first study’s purpose is to explore whether claustrophobia tendencies can predict spatial needs in a digital space.

### Methods

Three hundred and one U.S. residents (women: 58.1%) participated in the study through Prolific and Mechanical Turk. The sample size (*n* = 301) was determined to achieve 80% power to detect minimal effect sizes that are statistically significant (Cohen, 1992; Merkle et al., 2021). In the announcements to recruit participants, specific terms including claustrophobia and spatial constraints were not included, thereby allowing for the collection of unbiased samples. Following a brief review of an unrelated website, the participants (18–34: 59.8%; 35–54: 32.9%; 55+: 7.3%) completed a questionnaire that included measures on (1) claustrophobic tendencies (*α* = 0.95) using the statements (Appendix A) from Öst’s Claustrophobia Scale (2007) and (2) needs for digital space (*α* = 0.67)^1^ that involved six statements regarding various spatially constrained situations in digital environments (see the sample statements and rationale in Appendix A and Supplementary Information).

### Results and Discussion

Participants showed a moderate level of claustrophobic tendency (*M* = 3.97, *SD* = 1.36) and need for digital space (*M* = 4.70, *SD* = 1.06; Appendix B), which did not significantly differ by their age (*p* > 0.05; Appendix B).

Regression was used to test the proposed effects. For item-by-item correlation, Spearman was used as the data were taken from ordinal scales (Field and Miles, 2010).

#### Scale Correlation

The correlation between claustrophobic tendencies and the need for digital space was significant (*r_spearman_* = 0.37, *p* < 0.001). When compared with the two dimensions of need for digital space, claustrophobic tendencies were more closely correlated with the task-driven spatial needs (D4, D5, and D6; *r_speaman_* = 0.42, *p* < 0.001) than with the device-driven needs (D1, D2, and D3; *r_spearman_* = 0.11, *p* = 0.06). The result suggests that digital spatial needs are more closely associated with task-driven than device-driven needs.

Table 1 also shows that most items of the claustrophobic tendency scale were correlated predominantly with the task-driven needs, which focused on reading tasks. Only a few items were correlated with the device-driven needs, which measured spatial needs for screen space. These results suggest that claustrophobic tendencies might be more apparent when tasks require more cognitive attention (e.g., reading a lengthy document on a device) than when tasks are spatially constrained by device size (e.g., small screen space).

**Table 1:** Spearman correlation between claustrophobic tendency and need for digital space (Study 1).

Claustrophobic tendency	Need for digital space					
Item no.	D1	D2	D3	D4	D5	D6
C1	0.237^**^	0.256^**^	0.069	0.209^**^	0.177^**^	0.081
C2	0.002	0.109	0.043	0.120^*^	0.146^*^	0.312^**^
C3	−0.126^*^	−0.024	0.058	0.201^**^	0.254^**^	0.403^**^
C4	−0.159^**^	−0.072	0.018	0.181^**^	0.183^**^	0.313^**^
C5	−0.206^**^	−0.030	0.024	0.126^*^	0.252^**^	0.343^**^
C6	−0.104	0.083	0.089	0.252^**^	0.269^**^	0.372^**^
C7	−0.064	0.085	0.079	0.265^**^	0.310^**^	0.335^**^
C8	−0.026	0.106	0.094	0.159^**^	0.241^**^	0.307^**^
C9	−0.023	0.004	−0.014	0.168^**^	0.245^**^	0.372^**^
C10	−0.177^**^	0.049	0.060	0.213^**^	0.299^**^	0.418^**^
C11	−0.099	0.053	0.069	0.216^**^	0.285^**^	0.429^**^
C12	−0.011	0.116^*^	0.045	0.147^*^	0.205^**^	0.330^**^
C13	0.127^*^	0.147^*^	0.126^*^	0.160^**^	0.213^**^	0.128^*^
C14	−0.012	0.097	0.055	0.176^**^	0.254^**^	0.341^**^
C15	0.019	0.100	0.102	0.153^**^	0.205^**^	0.305^**^
C16	−0.021	0.054	0.091	0.178^**^	0.239^**^	0.296^**^
C17	−0.155^**^	0.007	0.062	0.140^*^	0.223^**^	0.401^**^
C18	0.308^**^	0.240^**^	0.091	0.083	0.121^*^	−0.048
C19	0.166^**^	0.263^**^	0.134^*^	0.226^**^	0.247^**^	0.155^**^

* Statistically significant at the *p* < 0.05 level (2-tailed).

** Statistically significant at the *p* < 0.01 level (2-tailed).

#### Need for Digital Space

**Figure 1:**
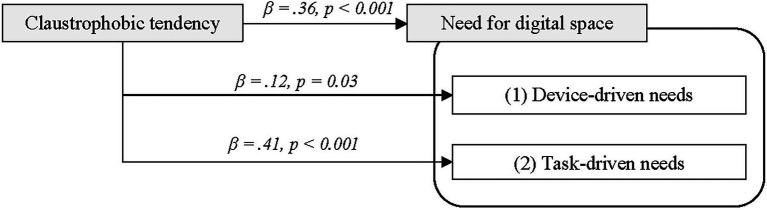
The effect of claustrophobic tendency on need for digital space (Study 1).

The regression results reveal that claustrophobic tendencies predicted need for digital space (*β* = 0.36, *p* < 0.001; Table 2). That is, if participants typically were sensitive to spatially constrained physical surroundings, such as a small confined or crowded space, they were also sensitive toward spatial constraints in digital interfaces, supporting H1 (Figure 1). The effect of claustrophobic tendencies was also significant on (1) the device-driven needs (*β* = 0.12, *p* = 0.03) and (2) the task-driven needs (*β* = 0.41, *p* < 0.001).

**Table 2:** The impact of claustrophobic tendency on need for digital space.

Predictor variables		Need for digital space (D1–6)	Device-driven need (D1, 2, and 3)	Task-driven need (D4, 5, and 6)	Spatial constraint
Study 1	Claustrophobic tendency	0.36 (0.04)^***^	0.12 (0.05)^*^	0.41 (0.06)^***^	–
Study 2	Claustrophobic tendency	0.50 (0.50)^***^	0.24 (0.06)^**^	0.55 (0.07)^***^	0.60 (0.07)^***^
	Grid complexity (0 = simple; 1 = complex)	–	–	–	0.22 (0.14)^**^
	Claustrophobic tendency^*^grid complexity	–	–	–	–0.19 (0.10)^*^

Standard errors in parentheses.

* Statistically significant at *p* < 0.05.

** Statistically significant at *p* < 0.01.

*** Statistically significant at *p* < 0.001.

In summary, the results show a strong association between claustrophobic tendencies and need for digital space; thus, H1 is supported. The stronger effect with task-driven needs implies that digital spatial needs and discomfort are particularly stronger in heavy reading or cognitively loaded occasions.

## Study 2: Perceived Spatial Constraints

This study was designed to test whether claustrophobic tendencies and digital spatial needs similarly influence users’ perceived spatial constraints on a website.

### Methods

Using the same announcement from Study 1, I recruited participants (*n* = 170 U.S. residents; women: 58%) through Mechanical Turk. The participants (18–34: 42.9%; 35–54: 41.2%; 55+: 15.9%) were randomly assigned to one of the two grid complexity conditions (simple vs. complex). Grid complexity differed by the level of spatial constraint and by the number of columns on a test product webpage, similar to Couper et al. (2013). Participants in the simple grid condition viewed a product webpage, in which the product information was displayed in *one column* (*n* = 91), while those in the complex grid condition viewed the same product in a *two-column* (*n* = 79) layout. The sample size was determined to achieve 80% power to detect medium effect sizes that are statistically significant (Cohen, 1992).

In each session, participants read a shopping scenario and reviewed multiple options of headset with a microphone for work, using their PC, which I screened through the user agent (UA) based device screening option in Qualtrics. They then completed the same questions used in Study 1 (Appendix A): (1) claustrophobic tendency (*α* = 0.96) and (2) need for digital space (*α* = 0.70). I also measured (3) perceived spatial constraint (*α* = 0.61)^2^ based on the existing studies with a focus on spatial crowding dimensions (Machleit et al., 1994, 2000; see Appendix A for details).

### Results and Discussion

The average claustrophobic tendency (*M* = 4.58, *SD* = 1.38) and need for digital space were moderate (*M* = 5.10, *SD* = 1.01). Claustrophobic tendency did not differ by age (*p* = 0.37) while need for digital space did between some groups (Appendix B). The same tools used in Study 1 were employed for the correlation and proposed effects.

#### Manipulation Check

The grid complexity manipulation was implemented successfully; ANOVA results demonstrated that participants who viewed a webpage with a complex grid perceived the webpage to be more spatially constrained (*M_complex_* = 5.03, *SD_complex_* = 1.08) than those who viewed one with a simple grid structure (*M_simple_* = 4.63, *SD_simple_* = 0.99; *F*(1, 168) = 6.29, *p* = 0.01).

#### Scale Correlation

The correlation between claustrophobic tendencies and need for digital space was also significant (*r_spearman_* = 0.55, *p* < 0.001). The correlations were also significant when tested with the averages of both dimensions of need for digital space separately; claustrophobic tendencies had a significant correlation with the device-driven (*r_spearman_* = 0.25, *p* < 0.001) and task-driven spatial needs (*r_spearman_* = 0.59, *p* < 0.001).

The items in the claustrophobic tendency scale were more closely associated with the task-driven items, similar to the findings of Study 1 (Table 3), and this consistency across both studies imply that claustrophobic tendencies in a digital space are likely to appear with cognitively demanding tasks.

**Table 3:** Spearman correlation between claustrophobic tendency and need for digital space (Study 2).

Claustrophobic tendency	Need for digital space					
Item no.	D1	D2	D3	D4	D5	D6
C1	0.388^**^	0.288^**^	0.222^**^	0.136	0.283^**^	0.240^**^
C2	0.051	0.200^**^	0.135	0.278^**^	0.434^**^	0.419^**^
C3	−0.049	0.149	0.108	0.293^**^	0.395^**^	0.454^**^
C4	0.047	0.163^*^	0.053	0.198^**^	0.371^**^	0.310^**^
C5	0.007	0.101	0.077	0.241^**^	0.417^**^	0.496^**^
C6	−0.137	0.155^*^	0.027	0.292^**^	0.371^**^	0.432^**^
C7	0.075	0.269^**^	0.153^*^	0.321^**^	0.467^**^	0.397^**^
C8	0.123	0.282^**^	0.146	0.344^**^	0.434^**^	0.426^**^
C9	0.024	0.143	0.047	0.242^**^	0.424^**^	0.441^**^
C10	−0.046	0.147	0.176^*^	0.279^**^	0.450^**^	0.466^**^
C11	−0.049	0.139	0.129	0.215^**^	0.445^**^	0.428^**^
C12	0.049	0.279^**^	0.264^**^	0.328^**^	0.475^**^	0.411^**^
C13	0.150	0.127	0.200^**^	0.157^*^	0.383^**^	0.294^**^
C14	0.142	0.224^**^	0.172^*^	0.262^**^	0.503^**^	0.506^**^
C15	0.072	0.159^*^	0.196^*^	0.210^**^	0.386^**^	0.400^**^
C16	0.072	0.191^*^	0.218^**^	0.182^*^	0.525^**^	0.450^**^
C17	−0.030	0.129	0.095	0.237^**^	0.411^**^	0.480^**^
C18	0.324^**^	0.320^**^	0.215^**^	0.170^*^	0.267^**^	0.189^*^
C19	0.207^**^	0.333^**^	0.204^**^	0.262^**^	0.342^**^	0.344^**^

* Statistically significant at the p < 0.05 level (2-tailed).

** Statistically significant at the p < 0.01 level (2-tailed).

#### Need for Digital Space

Similar to Study 1, as shown in Table 2, participants with elevated claustrophobic tendencies would exhibit an elevated need for digital space (*β* = 0.50, *p* < 0.001), hence supporting H1. As in Study 1, the effect of claustrophobic tendencies was significant with the two dimensions: device-driven needs (*β* = 0.24, *p* = 0.002) and task-driven needs (*β* = 0.55, *p* < 0.001; see Table 3 for details).

#### Spatial Constraint

**Figure 2:**
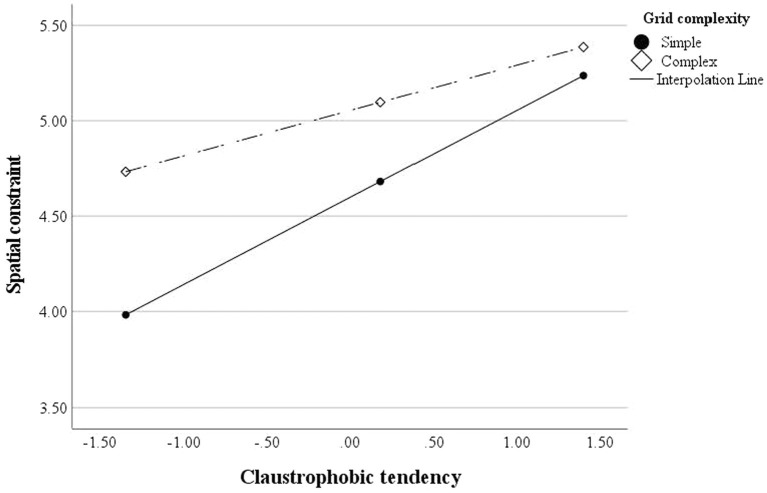
Interaction effect between grid complexity and claustrophobic tendency (Study 2). The simple grid condition was coded as 0, and the complex grid condition was coded as 1. The claustrophobic tendency was mean-centered (*M* = 4.58). The interaction effect was significant (*p* = 0.03).The results were analyzed based on Krishna’s spotlight analysis (i.e., conditional effects; Krishna, 2016) using PROCESS Model 1 (50,000 bootstrap samples; Hayes, 2018).

The complex grid condition (*β* = 0.22, *p* = 0.001) and elevated claustrophobic tendencies (*β* = 0.60, *p* < 0.001; mean-centered on *M* = 4.58) led to stronger spatial constraints. The grid conditions had a significant interaction effect with claustrophobic tendencies; viewing a complex grid webpage led to weaker spatial constraints with a lower claustrophobic tendency (*β* = −0.19, *p* = 0.03), supporting H1 and H2. In other words, although participants considered the complex webpage to be more spatially constrained, this effect was more apparent among those with low and moderately high claustrophobic tendencies. A follow-up spotlight analysis for conditional effects (Krishna, 2016) also confirms the interaction effect (Figure 2), and the effect with the original data (not mean-centered) was similar (*p* = 0.03; see Appendix C).

In a separate regression analysis, the complex grid (*β* = 0.43, *SE* = 0.11, *p* < 0.001) and stronger need for digital space (*β* = 0.18, *SE* = 0.15, *p* = 0.01; mean-centered on *M* = 5.10) also led to stronger spatial constraint. However, the effect of need for digital space did not significantly differ by grid complexity (*β* = −0.14, *SE* = 0.15, *p* = 0.16).

Between the two dimensions of need for digital space, a separate test shows that *task-driven* need had a significant interaction effect with grid complexity on spatial constraint (*β* = −0.22, *SE* = 0.11, *p* = 0.03), suggesting that viewing a complex webpage led to weaker spatial constraint with low task-driven need. The device-driven need, however, had no significant interaction effect with grid complexity (*p* = 0.80) and had no significant main effect on spatial constraint (*p* = 0.13), while grid complexity did (*β* = 0.18, *SE* = 0.16, *p* = 0.02). Thus, similar to Study 1, spatial constraints in digital environments may be stronger with cognitively demanding tasks than with small screen sizes.

Overall, the results indicate that both claustrophobic tendencies and the need for digital space are not only associated with each other but also impact online users’ perceived spatial constraints in the digital space, particularly more so with the complex interface design and task-driven spatial needs.

## General Discussion

There are several important theoretical and practical implications of the results, which are addressed in the following subsections.

### Theoretical Contributions

The findings confirm that claustrophobic tendencies can be used to predict the level of spatial needs on digital devices. People with elevated claustrophobic tendencies in a physical space (e.g., a retail store) are likely to feel more sensitive toward the size of digital space (e.g., an e-commerce site), show a stronger preference for a larger digital space (e.g., a larger screen space), and experience more spatial constraints. The stronger correlation between claustrophobic tendencies and the task-driven need for digital space also implies that digital spatial constraints are more closely linked to task-related visual and spatial sensory cues rather than the device-driven spatial needs. The results are also in line with the literature on spatial constraints (Eroglu et al., 2001, 2005; Sohn et al., 2017), visual–spatial ability (Jones et al., 2008), and digital reading (Mangen et al., 2019).

Additionally, the study provides additional insights into online users’ spatial needs. The findings introduce the potentially crucial role of online users’ claustrophobic tendencies in assessing their similar needs in a digital space. They expand the scope of the extant research on digital user experiences (U/X), which typically focused on investigating whether the spatial perception triggered by *design variations* (e.g., text spacing; grid complexity) affects user experiences (Couper et al., 2013; Liao and Keng, 2013; Yu and Kong, 2016; Miniukovich et al., 2017).

Lastly, the results present a novel way to use a conventional psychology theory (i.e., claustrophobia) to understand technology-driven consumer behavior. Although claustrophobia scales have been used to medically diagnose claustrophobic symptoms; the study demonstrates that these measures can be also used to understand non-diagnosed people’s spatial perception, which may influence their overall experiences and purchase decisions.

### Practical Implications

The findings suggest several implications for research and business practices in digital marketing, online retailing, and U/X design. Beyond the traditional design factors, individual users’ claustrophobic tendencies need to be considered when testing digital content design. Perhaps, a digital version of the claustrophobic tendency scale could be developed for web usability tests, measuring not only device- or task-driven needs, but also design-related spatial needs in web interfaces or even in virtual reality (e.g., Metaverse) or gaming.

In addition, current device settings should improve to support users’ varying spatial needs while maintaining essential features. Although device manufacturers have effectively responded to consumers’ need for larger screen sizes, the latest smartphone models with larger screens (e.g., 7 inches or larger) became too bulky to carry. Recently, Samsung successfully resolved the issues, using foldable screen technologies, while still providing a similar area of screen space (Cipriani, 2021). The brand’s new flip smartphone (Galaxy Z Flip 3)^3^ folds in half to fit more easily in a pocket than the existing phablet-style smartphones (e.g., iPhone 13) without sacrificing its screen space. Likewise, more manufacturers need to think outside the box to create better solutions that address the demand for larger digital spaces without compromising other features.

Furthermore, researchers and business practitioners should pay attention to the point that digital content may require more cognitive resources, particularly among people with elevated claustrophobic tendencies. Although further research is needed to verify what really drives the potential cognitive load, the findings imply that users may feel more uncomfortable reading cognitively demanding content (e.g., books, manuals, and lengthy legal documents) on digital devices. One of the factors that might drive the discomfort maybe the size of the reading space. Most e-book devices (e.g., Kindle) show content on a single-page view, which adds more spatial constraints to users’ cognitive load. Second factor can be the limited access to most spatial cues that would be typically available when reading physical books. For instance, people reading an e-book cannot locate specific content just by flipping pages, making it difficult to correctly find events in the story (Mangen et al., 2019). Instead, they have to cognitively process related information by reading page numbers or use a progress bar to understand how much they have read, increasing their cognitive load even further. Thus, even if multi-page view is allowed, not having such full spatial access to the book can still increase the cognitive load and spatial discomfort, increasing a “*desire to escape*” (O’Reily, 2011, p. 5) from the virtual surroundings. Therefore, further research is required to verify what kind of interfaces can reduce the expected cognitive load and spatial discomfort.

### Limitations and Future Research

The study presents an innovative way of using the conventional claustrophobic measure to assess digital spatial perceptions. While the findings offer important new insights, they have a few limitations. As briefly suggested, an expanded scale may be necessary to assess the need for digital space in more comprehensive contexts to accommodate not only web interfaces but also virtual or augmented reality interfaces. Although the claustrophobia scale used in my study was designed to measure space-related anxiety levels, other types of anxiety measures can be further explored in future research. Moreover, future research may want to consider testing additional factors, such as screen size, size of visual span, and scroll ability, to verify more specific effects.

In terms of generalizability, findings might be limited to the measures and/or the shopping scenarios tested in the studies. Additionally, using online subject panels, such as MTurk and Prolific, could involve a selection bias since the panels attract people who may be familiar with digital media. The device type that they used to complete the study could be their preferred device, and screening mobile users might have led to a relatively higher proportion of people with stronger claustrophobic tendencies, who are more likely to opt for larger devices.

Overall, the study is very timely as digital interfaces are now advancing to more comprehensive and full-scale spatial experiences through VR and AR, beyond traditional web interfaces. The findings are novel and open a new area for researchers to further verify claustrophobic effects on digital users’ spatial needs and perceptual experiences.

## Data Availability Statement

The original contributions presented in the study are included in the article/Supplementary Material, further inquiries can be directed to the corresponding author.

## Ethics Statement

The studies involving human participants were reviewed and approved by Rochester Institute of Technology. The participants provided their written informed consent to participate in this study.

## Author Contributions

The author confirms being the sole contributor of this article and has approved it for publication.

## Funding

This work was mainly supported by FEAD grant 2020–2021 from Rochester Institute of Technology.

## Conflict of Interest

The author declares that the research was conducted in the absence of any commercial or financial relationships that could be construed as a potential conflict of interest.

## Publisher’s Note

All claims expressed in this article are solely those of the authors and do not necessarily represent those of their affiliated organizations, or those of the publisher, the editors and the reviewers. Any product that may be evaluated in this article, or claim that may be made by its manufacturer, is not guaranteed or endorsed by the publisher.

## Footnotes

^1^α of 0.6–0.7 is an acceptable level of reliability, and 0.8 or greater indicates a very good level (Ursachi et al., 2015).

^2^α of 0.6–0.7 is an acceptable level of reliability, and 0.8 or greater indicates a very good level (Ursachi et al. 2015).

^3^Galaxy Z Flip 3 product page: https://www.samsung.com/us/smartphones/galaxy-z-flip3-5g.
