# Supplementary material for: The Effect of Claustrophobic Tendencies on Digital Spatial Preferences
Source: Front Psychol. 2022 Jun 23;13:874765. doi: 10.3389/fpsyg.2022.874765 (PMC9260385; doi:10.3389/fpsyg.2022.874765)
Supplement: Supplementary file 1 [file Data_Sheet_1.docx]

**Appendices**

**Appendix A. Scales**

| **Variables** | **Scale** | **Sample Statements** | **Reference** |
| --- | --- | --- | --- |
| Claustrophobia tendency | 1 = none (not anxious at all), 7 = very anxious | 1. Standing in such a crowd that you cannot move at all. 2. Being in a small room without windows. 3. Trying out clothes in a small fitting room with the door locked. 4. Sitting by the window in the middle of an airplane. 5. Riding a small elevator by yourself. 6. Trying out garments that are narrow in the neck. 7. Sitting in the middle of a crowded cinema or theatre. 8. Sitting by the window in a crowded bus with someone in the aisle seat. 9. Being in a windowless room in the basement. 10. Going in the back seat of a two-door car. 11. Walking through a narrow passage. 12. Going in the back seat of a small car with two other people. 13. Riding a small elevator with the maximum number of passengers. 14. Standing in a crowded bus that stops at a red light. 15. Being outdoors in a fog when you only can see a few yards in front of you. 16. Going in a sleeper car with two fellow passengers. 17. Entering a windowless lavatory and closing the door behind you. 18. Getting stuck between two floors in a small elevator. 19. The lock of the door to a small windowless lavatory has jammed. | Öst, 2007 |
| Need for digital space^i^ | 1 = strongly disagree, 7 = strongly agree | 1. For me, it's usually easier to complete my work-related tasks (e.g., preparing an Excel sheet; editing photos or documents) on a larger monitor than on a tablet or smartphone. 2. I often feel frustrated when I have to use a small device (e.g., smartphone) to complete a task 3. I prefer using multiple monitors over a single monitor for my work-related tasks. 4. I don't like reading a book on a digital device. 5. I feel cramped when reading a book or a lengthy document on a digital device 6. When reading an important document, I tend to print it to review its details more carefully. |  |
| Spatial constraint^ii^ | 1 = strongly disagree, 7 = strongly agree | - The webpage design seemed very spacious.* - I felt cramped reviewing the information on the webpage. - The webpage design had an open, airy feeling to it.* - The webpage design felt confining to me. | Machleit et al., 1994; Machleit et al., 2000 |

*Reverse coded

**i. Need for digital space**

The statements for the need for digital space, were created based on two dimensions of digital user experiences, device-driven and task-driven spatial needs, suggested by the literature. Several studies (Chae and Kim, 2004; Kim and Sundar, 2014; Schmidt and Maier, 2019) indicate that digitally dense interfaces due to smaller screen sizes lead to negative emotional or behavioral downstream consequences, suggesting that digital spatial needs can be driven by a device feature, namely screen size. Another stream shows that spatial discomfort may also occur owing to the type of task, such as reading information that requires much attention. Reading performance is closely associated with readers’ visual-spatial ability (Jones et al., 2008) and visual space around the text (Paterson and Jordan, 2010; Yu et al., 2010). Between printed and digital text, processing digitally displayed information may be more visually demanding than processing printed information because the digital text does not provide spatial cues to process text information as the print text does (e.g., flipping pages; manipulation of a printed book) and is harder to correctly locate events in the space of the text and temporality of the story (Mangen et al., 2019). Likewise, not having sufficient spatial cues and visual information in digital interfaces may be why printed books are still more popular^[[1]](#footnote-1)^ than digital books (Richter, 2021). This suggests that spatial needs may differ by the type of users’ tasks. Based on these research streams, the author incorporated the potential two dimensions of digital spatial needs (i.e., device-driven and task-driven needs) into the needs for digital space measurements; the first three statements (i.e., D1, D2, and D3) focus on the device-driven needs, and the next three statements (i.e., D4, D5, and D6) are designed to measure task-driven needs (see Supplementary Information for details).

**Validity check (Studies 1 and 2)**

|  | | | Factor loading | | | |
| --- | --- | --- | --- | --- | --- | --- |
|  |  |  | Study 1 | | Study 1 | |
| Scale | | Item | Factor 1*  (λ = 2.34) | Factor 2*  (λ = 1.27) | Factor 1*  (λ = 2.44) | Factor 2*  (λ = 1.18) |
| Need for digital space  (Study 1: α = .67;  Study 2: α =.70 ) | Device-driven need | D1 | - | .79 | - | .83 |
|  |  | D2 | - | .78 | .35 | .57 |
|  |  | D3 | - | .55 | - | .74 |
|  | Device-driven need | D4 | .79 | - | .83 | - |
|  |  | D5 | .84 | - | .84 | - |
|  |  | D6 | .74 | - | .65 | - |

*Coefficients below .30 were suppressed.

**ii. Spatial constraint**

From Machleit et al. (1994), I referred to the items on perceived crowding, also used in Machleit’s other study (2000), with a focus on the spatial crowding dimension such as “The store seemed very spacious,” “1 felt cramped shopping in the store,” “The store had an open, airy feeling to it,” and “The store felt confining to shoppers,” which were modified for digital contexts.

**Appendix B. Descriptive statistics and age effects**

**Study 1.** Participants’ claustrophobic tendency ranged from 1.00 to 6.84, and the average claustrophobic tendency was near the midpoint (*M* = 3.97, *SD* = 1.36, variance = 1.85), which did not significantly differ by the participants’ age (*F*(1, 295) = 1.44, *p* = .21). The participants’ need for digital space showed a similar range from 1.67 to 6.84 (*M* = 4.70, *SD* = 1.06, variance = 1.12). The need for space also did not differ significantly by age (*F*(1, 295) = 1.91, *p* = .09). These data suggest that regardless of age, the randomly recruited general population has a moderate level of claustrophobic tendencies and need for digital space.

**Study 2.** Participants’ claustrophobic tendency was between 1.00 to 7 on the seven-point scale, and their average claustrophobic tendency was moderate (*M* = 4.58, *SD* = 1.38, variance: 1.90). Their needs for digital space ranged from 2.00 to 7.00 on the seven-point scale, and its average was 5.10 (*SD* = 1.01, variance = 1.03). Similar to Study 1, the data suggest a moderate level of claustrophobic tendencies and need for digital space among the general population.

Age did not have a significant effect on claustrophobic tendency (*F*(1, 164) = 1.08, *p* = .37). However, the need for digital space was significantly higher among younger groups (*F*(1, 164) = 2.74, *p* = .02), particularly between the youngest group (age 18–24) and older groups aged 35–44 (*p* = .02), 45–54 (*p* = .01), and 55–64 (*p* = .03), while having no significant difference from the oldest group (age 65–74; *p* = .40). Even so, when tested separately by each dimension of need for digital space, age had no significant effect on both device-driven (*p* = .052) and task-driven needs (*p* = .11).

**Appendix C. Interaction effect between grid complexity and claustrophobic tendency (Study 2)**


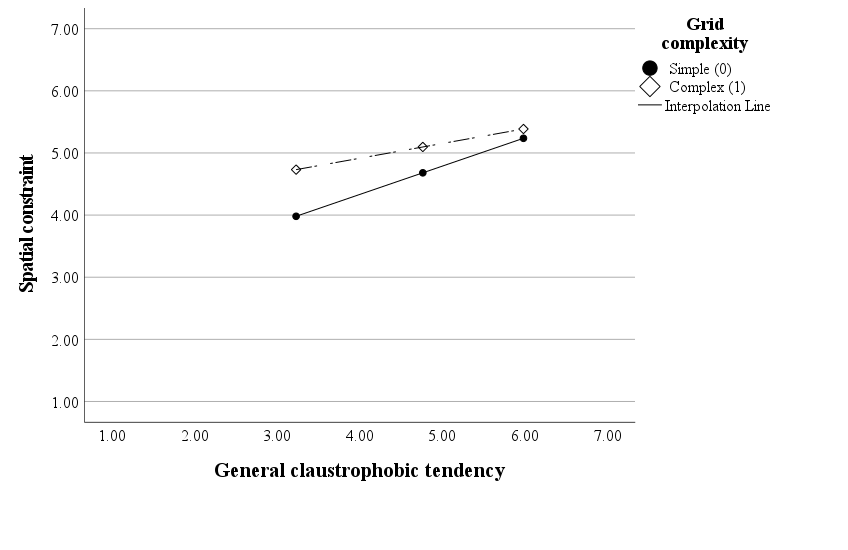


**Claustrophobic tendency**

The simple grid condition was coded as 0, and the complex grid condition was coded as 1. The claustrophobic tendency was not mean-centered, and the interaction effect was significant (*p* = .03). The results were analyzed based on Krishna’s spotlight analysis (i.e., conditional effects; Krishna, 2016) using PROCESS Model 1 (50,000 bootstrap samples; Hayes, 2018).

1. In 2020, 45% of the U.S. consumers purchased a print book while only 23% of them purchased an e-book (Richter, 2021). Even among university students, who are likely to be more exposed to e-books than older age groups, only 50.7% of them preferred e-books (Toor et al., 2021). [↑](#footnote-ref-1)
