# Supplementary material for: The Effect of Claustrophobic Tendencies on Digital Spatial Preferences
Source: Front Psychol. 2022 Jun 23;13:874765. doi: 10.3389/fpsyg.2022.874765 (PMC9260385; doi:10.3389/fpsyg.2022.874765)
Supplement: Supplementary file 2 [file Data_Sheet_2.docx]

**Supplementary Information**

**Need for digital space**

The purpose of this section is twofold: to detail how the need for digital space was operationalized and to elaborate on the rationale presented in Studies 1 and 2.

Operationalization began by reviewing the literature on user interfaces and visual information processing to understand potential spatial needs in digital space. Next, the overall digital browsing experiences were divided into (1) *device environments*, consisting of individual device features (e.g., screen size), and (2) *usage occasions*, which vary according to the purpose of a task (e.g., online shopping, reading, or viewing a video). Then, negative emotional and behavioral outcomes triggered by individual device features were identified, as were usage occasions in the context of digital space and/or spatial perceptions reported in the literature.

1. As for device environments, the literature on device-related effects in the context of spatial cues largely discusses the role of screen size, which often determines the size of users’ digital space. The related findings in the literature are shown in Table S1.

**Table S1. Device related findings**

| **Study** | **Related findings** |
| --- | --- |
| Chae and Kim (2004) | Both screen size and information structure influence users’ navigation behavior. |
| Dillon et al. (1990) | Larger displays seem to aid reading comprehension. |
| Kim and Sundar (2014) | Using a large (vs. small) mobile screen positively influences perceived ease of use. |
| Schmidt and Maier (2019) | Screen framing ratio and product orientation may bias product size assessment. |
| Sohn et al. (2017) | Perceived spatial crowding mediates the negative effects of perceived visual complexity on satisfaction among smartphone users. |

1. In terms of usage occasions, I focused on reading tasks due to their potential connections with space-related variables and spatial constraints in the literature. In studies on screen size (Chae and Kim, 2004; Dillon et al., 1990), screen size effects have been associated with the structure of the textual information and/or reading-related performance or otherwise moderated the screen size effects on task performance. Moreover, as shown in Table S2, many studies on spatial constraints have demonstrated that lower text information processing and lower reading-related task performance are downstream consequences of spatial constraints, thereby suggesting a close connection between spatial perception and reading.

**Table S2. Task-related findings**

| **Study** | **Related findings** |
| --- | --- |
| Chae and Kim (2004) | The effects of screen size on users’ navigation/perceptions may differ by task complexity. |
| Dillon et al. (1990) | Larger displays seem to help reading comprehension. |
| Kim and Sundar (2016) | Type of information processing and trust level sequentially mediate the effects of screen size on purchase intentions. |
| Jones et al. (2008) | Dyslexic (vs. non-dyslexic) readers showed decreased visual task performance |
| Mangen et al. (2019) | Reading a print pocket book (vs. Kindle) leads to better performance in locating events in the text and in the temporality of the story. |
| Paterson and Jordan (2010) | Increased letter spacing affects word identification. |
| Yu et al. (2010) | The visual layout is closely associated with reading performance. |

Based on the findings, the broad, initially proposed categories were refined into (1) *device-driven needs*, which are more likely to be associated with the size of screen space, and (2) *task-driven needs*, which are more likely to be linked to reading and/or other cognitively demanding tasks performed in digital space. Then, two sets of statements for each dimension were created, as shown in Appendix A, and a pretest was conducted for reliability with a smaller sample (*n* = 137). Regardless of the dimensions, the average individual responses ranged from 2.33 to 7.00 (*M* = 5.17; *SD* = 1.00; variance = .99), and the overall reliability (i.e., Cronbach’s alpha) of the six statements was .71.

For each dimension, the statements for task-driven needs showed stronger reliability (α = .72) than the ones for device-driven needs (α = .44). In the main studies, the reliability of the dimensions was consistent across both Studies 1 (α = .67; device-driven: α = .53, task-driven: α = .72) and 2 (α =.70; device-driven: α = .57, task-driven: α = .71). The validity check also confirms the two dimensions as key constructs (factors 1 and 2) as shown below.

**Table S3. Validity check (pre-test)**

|  | |  | Factor loading | |
| --- | --- | --- | --- | --- |
| Scale | | Item | Factor 1^*^  (λ = 2.50) | Factor 2^*^  (λ = 1.04) |
| Need for digital space  (α = .71) | Device-driven need | D1 | - | .93 |
|  |  | D2 | .53 | .33 |
|  |  | D3 | .58 | .41 |
|  | Task-driven need | D4 | .79 | - |
|  |  | D5 | .83 | - |
|  |  | D6 | .69 | - |

*Coefficients below .30 were suppressed.
